# Validation of prognostic indices in Egyptian Budd-Chiari syndrome patients: A single-center study

**DOI:** 10.3748/wjg.v23.i4.629

**Published:** 2017-01-28

**Authors:** Mohammad Sakr, Sara M Abdelhakam, Soheir A Elsayed, Enas H Allam, Amir M Farid, Waleed Abdelmoaty, Azza M Hassan, Mohamed Shaker, Mohamed El-Gharib, Ahmed Eldorry

**Affiliations:** Mohammad Sakr, Sara M Abdelhakam, Soheir A Elsayed, Enas H Allam, Amir M Farid, Waleed Abdelmoaty, Department of Tropical Medicine, Faculty of Medicine, Ain Shams University, Cairo 11341, Egypt; Azza M Hassan, Department of Community, Environmental and Occupational Medicine, Faculty of Medicine, Ain Shams University, Cairo 11341, Egypt; Mohamed Shaker, Mohamed El-Gharib, Ahmed Eldorry, Department of Radiodiagnosis and Interventional Radiology, Faculty of Medicine, Ain Shams University, Cairo 11341, Egypt

**Keywords:** Budd-Chiari syndrome, Prognostic indices, New Clichy score, One-year survival, Transjugular intrahepatic portosystemic shunt

## Abstract

**AIM:**

To compare predictive ability of Budd-Chiari syndrome (BCS) prognostic indices (PIs) for one-year survival and Transjugular intrahepatic portosystemic shunt (TIPS) patency.

**METHODS:**

This retrospective study enrolled 194 Egyptian patients with primary BCS who presented to the Budd-Chiari Study Group of Ain Shams University Hospital. Calculation of the available PIs was performed using Child-Pugh and model for end-stage liver disease scores, BCS-specific PIs (Clichy, New Clichy and Rotterdam) for all patients, and BCS-TIPS PI only for patients who underwent TIPS. The overall one-year survival rate and the one-year shunt patency rate for TIPS were reported.

**RESULTS:**

The overall one-year survival rate was 69.6%, and the New Clichy PI revealed the best validity for its prediction at a cut-off value of 3.75, with sensitivity and specificity of 78% and 73.3%, respectively [area under receiver operating characteristic curve (AUC) = 0.806]. The one-year survival rate post-TIPS was 89.7%, and the BCS-TIPS score demonstrated validity for its prediction at a cut-off value of 3.92 (sensitivity and specificity were 71.4% and 64.5%, respectively) (AUC = 0.715). Logistic regression analysis revealed that the New Clichy PI (*P* = 0.030), high serum total bilirubin (*P* = 0.047) and low albumin (*P* < 0.001) were independent factors for predicting mortality within one year. The one-year shunt patency rate in TIPS was 80.2%, and none of the PIs exhibited significant validity for its prediction.

**CONCLUSION:**

The New Clichy score could independently predict the one-year survival in Egyptian BCS patients.

**Core tip:** We analyzed the predictive ability of Budd-Chiari syndrome (BCS) prognostic indices (PIs) for one-year overall survival and transjugular intrahepatic portosystemic shunt (TIPS) patency rate in 194 Egyptian patients. Calculation of the available PIs was performed using Child-Pugh and model for end-stage liver disease scores, BCS-specific PIs (Clichy, New Clichy and Rotterdam) for all patients, and BCS-TIPS PI only for patients who underwent TIPS. We found that the New Clichy score independently predicted one-year survival in Egyptian BCS patients.

## INTRODUCTION

Budd-Chiari syndrome (BCS) is caused by hepatic venous outflow obstruction from the small hepatic veins (HVs) to the site of entry of the inferior vena cava (IVC) into the right atrium[1].

It is difficult to predict the prognosis of BCS patients because of the large variability in clinical presentation and disease course[2]. Little is known about factors that may help predict the survival of BCS patients[3], and various trials were done to determine parameters that might predict the prognosis in these patients[4].

Several scores were evaluated in BCS, including the Child-Pugh score, the model for end-stage liver disease (MELD) score and several BCS-specific prognostic indices (PIs), including the Clichy PI, the Rotterdam score, the New Clichy PI and the BCS-TIPS score[5]. These scores contain clinical and laboratory parameters and can be used to stratify BCS patients, however, their use for the management of an individual patient is still controversial[6].

Patient characteristics, etiological factors, and treatment outcomes have changed since these indices were elaborated, and comparability between studies from different centers is crucial for rapid advances in BCS[7]. Comparability relies on adjustments for baseline characteristics and requires the availability of a single, validated and widely accepted PI. However, an accurate PI to make therapeutic decisions in individual patients has not been established[8,9].

The aim of the present study was to compare the predictive ability of the available PIs for BCS for the one-year overall survival and the one-year shunt patency rate of transjugular intrahepatic portosystemic shunt (TIPS) in Egyptian patients.

## MATERIALS AND METHODS

This retrospective cohort study enrolled 194 Egyptian patients with primary BCS who presented to the Budd-Chiari Study Group (BCSG), Tropical Medicine Department of Ain Shams University Hospital (Cairo, Egypt) between November 2005 and December 2014. The study protocol was approved by the Research Ethical Committee of Faculty of Medicine, Ain Shams University according to the ethical guidelines of the 1975 Declaration of Helsinki.

Patients with any other concomitant cause of liver disease (*e.g*., viral, autoimmune or metabolic), secondary BCS or hepatocellular carcinoma were excluded.

The following patient medical records and databases were reviewed: (1) clinical data; (2) laboratory investigations: CBC, liver profile, coagulation profile, viral markers (HBsAg, HBcAb, HCV Ab) using the enzyme-linked immunosorbent assay (ELISA) technique; (3) thrombophilia workup to clarify the underlying etiology of BCS as follows: Antinuclear antibodies, anti-β2 glycoprotein-1, anticardiolipin antibodies IgM and IgG were measured by ELISA technique and Lupus anticoagulant was measured by coagulation-based functional assay to diagnose antiphospholipid syndrome (APS). Protein C, S and antithrombin III were measured by coagulation-based functional assay to diagnose protein C, S, or antithrombin III deficiency. Genotyping of factor V Leiden G1691A, prothrombin G20210A, and methylene tetrahydrofolate reductase (MTHFR) C677 were performed *via* real-time PCR and fluorescence melting curve detection analysis to diagnose mutations. Janus tyrosine kinase-2 (JAK II) *V617F* mutation was detected by PCR and/or a bone marrow biopsy to diagnose myeloproliferative disorder (MPD). Flow cytometry for CD55 and CD59 was done to diagnose paroxysmal nocturnal hemoglobinuria[10]; and (4) radiological assessment using abdominal duplex ultrasonography (US) to assess the patency of the HVs, the portal vein (PV), and the IVC. Abdominal multi-slice computed tomography, magnetic resonance imaging and/or MR venography were performed when indicated to confirm all diagnoses and assess vascular anatomy.

Calculation of available BCS PIs was performed for all patients; from their data at initial presentation; as follows: (1) Modified Child-Pugh score: The sum of the scoring points from the five parameters [ascites (none = 1 point, moderate = 2 points, severe = 3 points), serum bilirubin (< 2 mg/dL = 1 point, 2-3 mg/dL = 2 points, > 3 mg/dL = 3 points), albumin (> 3.5 g/dL = 1 point, 2.8-3.5 g/dL = 2 points, < 2.8 g/dL = 3 points), hepatic encephalopathy (absent = 1 point, grades 1 and 2 = 2 points, grades 3 and 4 = 3 points), and prothrombin time International Normalized Ratio “PT INR” (< 1.7 = 1 point, 1.71-2.30 = 2 points, > 2.30 = 3 points)] corresponds to one of three groups (Child A = 5-6 points, Child B = 7-9 points, Child C = 10 or more points)[11]; (2) MELD score: 3.8 × (ln serum bilirubin mg/dL) + 11.2 × (ln INR) + 9.6 × (ln serum creatinine mg/dL) + 6.4[12]; (3) Clichy PI: (ascites score × 0.75) + (Pugh score × 0.28) + (age × 0.037) + (creatinine × 0.0036), where ascites was scored as absent, controlled with sodium restriction or diuretics or resistant to medical treatment (scored as 1, 2 or 3, respectively)[8]; (4) Rotterdam BCS index: 1.27 × encephalopathy + 1.04 × ascites + 0.72 × prothombin time + 0.004 × bilirubin, where ascites and hepatic encephalopathy were scored as present (1) or absent (0) and prothrombin time as higher (1) or equal/lower (0) than an INR of 2.3[3]; (5) New Clichy PI: 0.95 × ascites score + 0.35 × Pugh score + 0.047 × age + 0.0045 × serum creatinine + (2.2 × form III) - 2.6, where ascites was scored as in Clichy PI, and clinic-pathological form III (acute on top of chronic) was defined by the presence of at least one acute and one chronic feature and coded as 1 for patients with form III and 0 for the other patients[9]; and (6) BCS-TIPS PI (only for patients who underwent TIPS procedure): age × 0.08 + bilirubin × 0.16 + INR × 0.63[5].

The patterns of management were reported. All enrolled patients received anticoagulant therapy as early as possible after securing risky esophago-gastric varices in an attempt to reduce the risk of clot extension and new thrombotic episodes. Treatment of the underlying prothrombotic cause was also initiated concomitantly, *e.g*., folic acid supplementation for MTHFR mutation and diuretic therapy when indicated. Angioplasty and/or stenting were used in patients with partial or short segment occlusion of HVs and IVC to re-establish the physiological drainage of portal and sinusoidal blood. Patients with BCS who were non-responsive to medical treatment or who were not candidates for angioplasty/stenting (*i.e*., complete occlusion of all HVs with patent IVC and PV) were treated using TIPS to transform the portal system into an outflow tract. TIPS was also performed in patients with failed trials of HV stenting. Living donor liver transplantation (LDLT) was performed for patients with liver decompensation (because they would not benefit from TIPS) and for patients with failed TIPS. A mesoatrial shunt was performed to decompress the liver as a bridge to liver transplantation in patients who were not fit for radiological intervention[13,14].

The overall one-year survival rate was reported for all included patients. The one-year shunt patency rate was reported for patients who underwent the TIPS procedure.

### Statistical analysis

Data analysis was performed using SPSS (SPSS Inc. 2009. PASW Statistics for Windows, Version 18.0, Chicago, IL, United States). Quantitative variables are presented as the mean and standard deviation to describe the studied patients. Qualitative variables are presented as counts and percentages. Student’s *t*-test was used to compare quantitative variables between two independent groups. The Chi-square test was used to compare qualitative data between groups. The receiver operating characteristic (ROC) curve was used to measure the prognostic ability and determine the best cut-off value for different PIs, and logistic regression analysis was used to measure the independent effect of some variables on one-year patient survival. Kaplan-Meier survival analysis was performed to assess one-year survival and one-year shunt patency for patients. *P* value < 0.05 was considered statistically significant.

The statistical methods of this study were reviewed by Azza M Hassan, Lecturer of Community, Environmental and Occupational Medicine, Faculty of Medicine, Ain Shams University, Cairo, Egypt.

## RESULTS

### Characteristics of the study population, clinical and investigational data

The current study included 194 Egyptian patients with primary BCS. Their mean age was 28.79 ± 8.94 years, with female predominance (111/194, 57.2%).

The most common etiology of BCS in the current study was FVLM, which was found in 57 patients (29.4%), followed by MTHFR mutation in 48 patients (24.7%) and MPD in 43 patients (22.2%); twenty-nine patients of them (67.4%) were overt and 14 patients (32.6%) were occult. Forty patients (20.6%) had primary APS, and 19 patients (9.8%) had secondary APS. PC deficiency was present in 25 patients (12.9%); PS deficiency was present in 6 patients (3.1%), and anti-thrombin III deficiency was present in 18 patients (9.3%). Seven patients (3.6%) were idiopathic. Multiple etiologies were present in 73 patients (37.6%). Twenty-six patients (13.4%) did not complete the etiological panel.

The most common clinical presentations were hepatomegaly in 181 patients (93.3%) followed by ascites in 166 patients (85.6%), and abdominal pain in 163 patients (84%). Jaundice was present in 85 patients (43.8%). The chronic form of presentation was found in 135 patients (69.6%) and the acute/subacute form was found in 59 patients (30.4%).

Single HV occlusion was diagnosed in 7 patients (3.6%). Two HVs were occluded in 32 patients (16.5%), and three HVs were occluded in 155 patients (79.9%). The IVC was involved (occluded/attenuated/web) in 32 patients (16.5%), and PV thrombosis was diagnosed in 8 patients (4.1%).

Most patients were Child B (45.9%), followed by Child C (28.9%) and Child A (25.3%). We found that Rotterdam class III was the most common (72.2%), followed by class II and class I (15.5% and 12.4%, respectively). The following mean values of different prognostic scores in the included patients were observed: Child score: 8.34 ± 2.29, MELD: 12.25 ± 7.03, Clichy: 5.02 ± 1.14, New Clichy: 3.72 ± 1.44, Rotterdam: 2.27 ± 1.58 and BCS-TIPS score (calculated only for the 107 patients who underwent TIPS): 3.70 ± 0.88.

### Patterns of management and one-year survival

Patients were classified into two groups according to the patterns of management of BCS in the current study. (1) the interventional group included 131 patients (67.5%) who underwent interventional management and medical treatment. A total of 107 patients in this group underwent the TIPS procedure, 20 patients had HV angioplasty with stenting, one patient had angioplasty without stenting, one patient had LDLT, and two patients underwent mesoatrial shunt; and (2) the non-interventional group included 63 patients (32.5%) who were not suitable for any intervention and were treated only medically with anticoagulation and treatment for the underlying etiology with or without diuretics.

**Figure 1:**
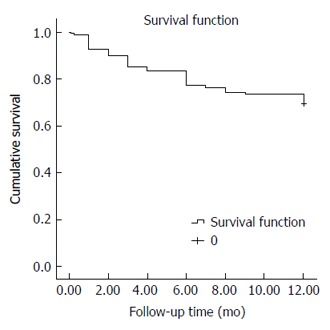
One-year survival function (time to death) of the included patients (Kaplan-Meier).

The overall one-year survival rate of the studied cohort was 69.6% (Total number of deaths by the end of first year: 59/194 patients). The estimated mean survival time was 9.84 mo (95%CI: 9.29-10.38) (Figure 1). The interventional group had a significantly better one-year survival rate than the non-interventional group. The one-year survival rates for both groups were 87.8% and 31.7%, respectively (number of deaths in the two groups was 16/131 and 43/63 patients, respectively) (*P* < 0.001).

Eleven (10.3%) of the 107 patients who underwent the TIPS procedure died by the end of the first year, and the one-year survival rate post-TIPS was 89.7%.

### Factors affecting overall one-year survival

The overall one-year survival rate was not significantly related to either age or gender. However, univariate analysis revealed that many factors significantly affected the one-year survival: the presence of genital and oral ulcers, history of DVT, use of hormonal therapy in females, acute and subacute presentations, presence of jaundice, hepatic encephalopathy, ascites and advanced ascites score. The non-survivor group exhibited significantly higher serum bilirubin with lower serum albumin compared to the survivor group. Poor prognosis and shortened survival was related to the presence of PV thrombosis and/or IVC occlusion (Table 1).

**Table 1:** Factors related to the one-year survival

	**Alive (*n* = 135)**	**Dead (*n* = 59)**	***χ*^2^/*t***	***P* value**
	***n* (%)**	***n* (%)**		
Age in years, mean ± SD	28.6 ± 8.47	29.22 ± 10.01	0.44	0.66
Gender				
Male	53 (39.3)	30 (50.8)	2.25	0.13
Female	82 (60.7)	29 (49.2)		
Genital/oral ulcer	2 (1.5)	6 (10.2)	7.84	0.01
History of DVT	13 (9.6)	17 (28.8)	11.56	0.001
Use of OCP^1^	15 (18.3)	11 (37.9)	5.25	0.02
Presentation				
Acute/subacute	31 (23)	28 (47.5)	11.64	0.001
Chronic	104 (77)	31 (52.5)		
Jaundice	45 (33.3)	40 (67.8)	19.81	< 0.001
Hepatic encephalopathy	14 (10.4)	24 (40.7)	23.94	< 0.001
Ascites	110 (81.5)	56 (94.9)	6.00	0.01
Ascites score^2^				
1	26 (19.3)	3 (5.1)	51.38	< 0.001
2	89 (65.9)	17 (28.8)		
3	20 (14.8)	39 (66.1)		
IVC (occluded/attenuated/web)	16 (11.9)	16 (27.1)	6.95	0.01
PV thrombosis	1 (0.7)	7 (11.9)	12.85	< 0.001
Total bilirubin, mean ± SD	2.15 ± 1.40	5.05 ± 4.93	4.44	< 0.001
Direct bilirubin, mean ± SD	0.90 ± 0.80	2.59 ± 2.94	4.34	< 0.001
Albumin, mean ± SD	3.46 ± 0.63	2.89 ± 0.65	5.75	< 0.001

1 Percentage was calculated among female patients;

2 Ascites score was calculated as (1): absent, (2): controlled with sodium restriction or diuretics, or (3): resistant to medical treatment. DVT: Deep venous thrombosis; OCP: Oral contraceptive pills; IVC: Inferior vena cava; PV: Portal vein.

### PIs and one-year survival

**Figure 2:**
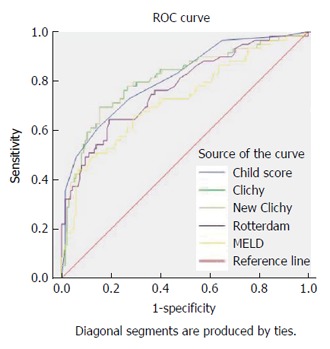
Receiver operating characteristic curve showing the validity of different prognostic indices (Child score, model for end-stage liver disease score, Clichy prognostic index, New Clichy prognostic index and Rotterdam index) for prediction of the one-year overall survival of the studied patients. MELD: Model for end-stage liver disease; PI: Prognostic index; ROC: Receiver operating characteristic.

All prognostic scores were significantly related to overall one-year survival, with significantly higher scores in patients who died (Table 2). Their area under ROC curves (AUC) exceeded 0.5. However, only three PIs exhibited significant validity and predictive ability regarding the overall one-year survival; which makes them useful for individual decisions in day-to-day practice because their AUC exceeded 0.8; these scores were New Clichy, Clichy and Child-Pugh scores. The New Clichy PI was the best factor (AUC = 0.806) at a cut-off value of 3.75, with sensitivity and specificity of 78% and 73.3%, respectively (Table 3 and Figure 2).

**Table 2:** Relation between different prognostic indices and the one-year survival rate

	**One-year survival**		***t***	***P* value**
	**Alive (*n* = 135)**	**Dead (*n* = 59)**		
	**mean ± SD**	**mean ± SD**		
Child score	7.54 ± 1.79	10.17 ± 2.28	7.85	< 0.001
MELD score	10.42 ± 5.44	16.42 ± 8.40	5.05	< 0.001
Clichy PI	4.64 ± 0.94	5.89 ± 1.09	8.12	< 0.001
New clichy PI	3.24 ± 1.18	4.82 ± 1.37	8.11	< 0.001
Rotterdam index	1.78 ± 0.88	3.41 ± 2.15	5.64	< 0.001
BCS-TIPS score^1^	3.61 ± 0.83	4.33 ± 0.97	2.99	0.003

1 Calculated only for 107 patients who underwent TIPS. MELD: Model for end-stage liver disease; PI: Prognostic index; BCS-TIPS score: Budd-Chiari syndrome-transjugular intrahepatic portosystemic shunt score.

**Table 3:** Diagnostic performance of prognostic indices for prediction of one-year survival among the studied patients

**Prognostic indices**	**Cut-off**	**Sensitivity**	**Specificity**	**AUC**	**SE**	***P* value**	**95%CI**
Child score	≥ 8.55	72.90%	72.60%	0.811	0.034	< 0.001	0.743-0.878
MELD score	≥ 11.59	69.50%	64.40%	0.723	0.042	< 0.001	0.641-0.805
Clichy PI	≥ 4.95	78.00%	70.40%	0.807	0.036	< 0.001	0.735-0.878
New clichy PI	≥ 3.75	78.00%	73.30%	0.806	0.036	< 0.001	0.735-0.878
Rotterdam index	≥ 1.94	71.20%	65.20%	0.771	0.038	< 0.001	0.696-0.845
BCS-TIPS score^1^	≥ 3.92	71.40%	64.50%	0.715	0.072	0.010	0.574-0.857

1 Calculated only for 107 patients who underwent TIPS. AUC: Area under the receiver operating characteristic (ROC) curve; MELD: Model for end-stage liver disease; PI: Prognostic index; BCS-TIPS score: Budd-Chiari syndrome-transjugular intrahepatic portosystemic shunt score; SE: Standard error.

**Figure 3:**
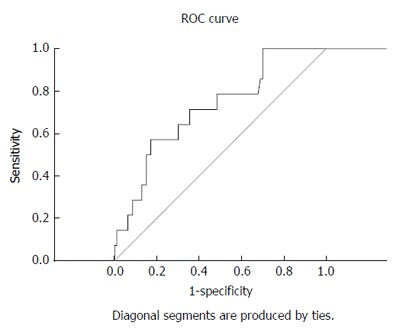
Receiver operating characteristic curve showing the validity of Budd-Chiari syndrome - transjugular intrahepatic portosystemic shunt score for prediction of the one-year survival among patients who underwent transjugular intrahepatic portosystemic shunt procedure (*n* = 107). ROC: Receiver operating characteristic.

The BCS-TIPS score exhibited validity for the prediction of one-year survival post-TIPS at a cut-off value of 3.92 (sensitivity and specificity were 71.4% and 64.5%, respectively) (AUC = 0.715) (Table 3 and Figure 3).

### Logistic regression analysis for factors affecting one-year survival

Multivariate logistic regression analysis for factors affecting the one-year survival for all studied patients revealed that New Clichy PI (*P* = 0.030), high serum total bilirubin (*P* = 0.047) and low serum albumin levels (*P* < 0.001) were independent factors for predicting mortality within one year (Table 4).

**Table 4:** Logistic regression analysis for factors affecting the one-year survival

	**B**	***P* value**	**OR (95%CI)**
New Clichy PI	0.291	0.030	1.338 (1.029-1.741)
Hepatic encephalopathy	0.731	0.118	2.077 (0.830-5.199)
Total bilirubin	0.194	0.047	1.214 (1.003-1.470)
Albumin	-0.857	< 0.001	0.424 (0.323-0.558)

B: Regression coefficient; PI: Prognostic index.

### Factors affecting one-year shunt patency in TIPS

Nineteen (19.8%) of the 96 TIPS patients who completed the one-year follow-up had occluded shunts at the end of the first year, and the one-year shunt patency rate of TIPS was 80.2%.

The one-year shunt patency was not related to any of the studied factors (demographic, clinico-laboratory, etiologic or PIs) (Table 5).

**Table 5:** Relation between different prognostic indices and the one-year shunt patency in transjugular intrahepatic portosystemic shunt patients

	**One-year shunt patency^1^**		***t***	***P* value**
	**Patent (*n* = 77)**	**Occluded (*n* =19)**		
	**mean ± SD**	**mean ± SD**		
Child score	7.67 ± 1.69	7.74 ± 2.00	0.15	0.89
MELD score	10.26 ± 5.32	10.56 ± 7.17	0.21	0.84
Clichy PI	4.70 ± 0.92	4.86 ± 1.06	0.65	0.52
New Clichy PI	3.32 ± 1.16	3.52 ± 1.34	0.65	0.52
Rotterdam index	1.79 ± 0.80	1.99 ± 1.05	0.91	0.37
BCS-TIPS score	3.57 ± 0.80	3.73 ± 1.03	0.69	0.49

1 Total transjugular intrahepatic portosystemic shunt (TIPS) patients was 107, among them eleven patients died and 96 patients completed the one-year follow up. MELD: Model for end-stage liver disease; PI: Prognostic index; BCS-TIPS score: Budd-Chiari syndrome-transjugular intrahepatic portosystemic shunt score.

## DISCUSSION

Several BCS-specific PIs and numerous clinical and laboratory parameters have been previously reported[15,16]. However, the predictive accuracy of these PIs remains insufficient for predicting the survival of BCS patients[5,6].

The current study compared the predictive ability of the available PIs for BCS for the overall one-year survival and the one-year shunt patency rate of TIPS in Egyptian patients.

The overall one-year survival rate was 69.6% in this study. We found a striking difference in survival at one year, which was higher in the group of patients who underwent intervention than patients who were unfit for intervention and managed only medically (87.8% *vs* 31.7%, respectively). This result is consistent with a systematic review of 79 studies discussing BCS survival by *Qi et al*[15] in 2015. The authors found that the median one-year survival rate was 93% (range: 80%-100%) in 9 previous studies performed on patients receiving interventional radiological treatments, and 68.1% (range: 14%-92%) in 6 studies that included patients receiving medical therapy alone. The survival figures may have been affected by differences in the selection criteria of the included BCS patients as well as their underlying disease etiologies which led to a strong influence on the expected natural history and outcome of the disease. In fact, BCS patients from different geographic regions tend to show distinct disease etiologies[17]. In particular, thromboses are more common in Western, whereas venous webs are more frequent in Eastern and Japanese BCS patients[18]. Our study of Egyptian BCS patients in 2011[19] as well as the current study indicated that FVLM and MTHFR mutation are the most commonly detected prothrombotic disorders in Egyptian BCS patients.

The survival of BCS patients demonstrated gradual improvement over time and a favorable prognosis[20,21]. The dramatic improvement in survival over years is easily explained by the increasing recognition of BCS, establishment of an effective treatment strategy, improvement in interventional radiological techniques, and advances in the management of portal hypertension-related complications[22,23]. Actually, the first year after diagnosis of BCS is a critical period and is related to longer term prognosis in those patients[15,20].

Neither age nor gender were related to prognosis in the univariate analysis in the current study, which is similar to previous reports[15]. We found that the presence of oral and genital ulcers and use of hormonal therapy were related to poor prognosis. This result is also consistent with previous reports[24,25]. The current study also revealed that acute and subacute presentations were linked to higher mortality rates compared to chronic presentation because the acute form may lead to fulminant hepatitis and acute liver cell failure, with a subsequent poor prognosis[26].

Patients who presented with PVT and/or IVC thrombosis exhibited higher mortality rates in the current study, which is consistent with DeLeve et al[10].

Univariate analysis demonstrated that all studied PIs (Child, MELD, Rotterdam, Clichy, New Clichy and BCS-TIPS scores) were significantly related to one-year survival in the current study and distinguished survivors from non-survivors. The survivor group of our patients exhibited lower values for all PIs than the non-survivor group. This result is consistent with the studies performed by Zhang et al[27] and Rautou et al[6] for all PIs except the BCS-TIPS score, which exhibited lower predictive ability in their studies. This discrepancy may be attributed to the different durations of follow-up and the different baseline criteria of the enrolled patients.

The Child, Clichy and New Clichy scores exhibited significant validity and predictive ability in the current study, which makes these scores useful for individual decisions in day-to-day practice (their AUC exceeded 0.8). This result is consistent with Rautou et al[6].

The New Clichy score in our patients exhibited the highest sensitivity (78%) and specificity (73.3%) at a cut-off value of > 3.75 for the prediction of one-year survival (AUC = 0.806), followed by the Clichy score, the Child score, the Rotterdam score, and the MELD score. In Chinese patients included in the study of Zhang et al[27], New Clichy score exhibited the highest specificity but lowest sensitivity (93.9% and 50%, respectively), and the Clichy score exhibited the highest sensitivity but lowest specificity (87.5% and 53.5%, respectively). These differences may be attributed to the fact that BCS exhibits characteristics that differ according to ethnic and geographical considerations[17]. In the study of Zhang et al[27], the authors didn’t mention any cut-off value for their PIs. However; through their ROC curve analysis, the New Clichy score AUC was the largest (0.776), and its Youden index was 0.44, indicating a high predictive value.

Multivariate logistic regression analysis in the current study revealed that the New Clichy PI (*P* = 0.030), high serum total bilirubin (*P* = 0.047) and low serum albumin levels (*P* < 0.001) were independent factors for predicting mortality within one year. Therefore, these factors were related to poor prognosis and outcome. Pavri et al[28] performed a multivariate analysis and demonstrated that increasing age, presence of cirrhosis at diagnosis and chronic kidney disease were significantly associated with poor prognosis, in contrast to bilirubin or other markers of liver disease severity, which were not related to prognosis. Fitsiori et al[29] found that the presence of ascites, elevated creatinine, Child-Pugh score and MELD score were predictors of prognosis. Different study designs, variable clinical presentations and laboratory parameters at diagnosis may explain the variability in the identification of prognostic factors for BCS.

The PIs, except the BCS-TIPS score, were developed in the pre-TIPS era, and these factors remain useful for the identification of patients with a poor prognosis on anticoagulation and supportive care who should be considered for TIPS. The introduction of TIPS dramatically improved prognosis[10]. The TIPS procedure remains an extremely effective therapy for eligible BCS patients with good survival rate[30]. The one-year survival rate post-TIPS in the current study was 89.7% and the BCS-TIPS score exhibited a good validity for its prediction. This is comparable to the study of Qi et al[31] and the meta-analysis of Zhang et al[21] which revealed a one-year survival rate of 83.8% and 87.3%, respectively.

The one-year shunt patency rate following TIPS was 80.2% in the current study. The patency rate was not related to any of the studied factors (demographic, clinico-laboratory, etiologic or PIs).

In conclusion, the New Clichy score could independently predict one-year survival in Egyptian BCS patients at a cut-off value of 3.75. None of the PIs exhibited significant validity for the prediction of one-year shunt patency of the TIPS procedure. Because BCS patients have different characteristics according to ethnic and geographical distribution; all PIs could be more or less good in stratifying patients in clinical trials. However, further extended studies are needed to clarify the possibility of using a single PI in the management of an individual patient.

## ACKNOWLEDGMENTS

The authors gratefully acknowledge members of the BCSG of Ain Shams University Hospital for their support.

## COMMENTS

### Background

Budd-Chiari syndrome (BCS) is a rare potentially life-threatening hepatic disorder caused by obstruction of hepatic venous outflow at any level from the hepatic venules to the right atrium. Little is known about factors that may predict the survival of BCS patients, and various trials were performed to determine parameters that may predict prognosis in these patients.

### Research frontiers

The authors analyzed the predictive ability of BCS prognostic indices (PIs) for the overall one-year survival and transjugular intrahepatic portosystemic shunt (TIPS) patency rate for 194 Egyptian patients. Calculation of the available PIs was performed which included Child-Pugh and model for end-stage liver disease scores, BCS specific PIs (Clichy, New Clichy and Rotterdam) for all patients, and BCS-TIPS prognostic index only for patients who underwent TIPS. They found that the New Clichy score could independently predict one-year survival in Egyptian BCS patients. The one-year shunt patency rate in TIPS was 80.2%, and none of the PIs exhibited significant validity for its prediction.

### Innovations and breakthroughs

This is the largest Egyptian study that addresses the predictive ability of BCS PIs for one-year overall survival and TIPS patency rate.

### Applications

This study may represent a future strategy for the use of the New Clichy score for predicting the one-year survival and making individual decisions in BCS.

### Terminology

New Clichy score = 0.95 × ascites score + 0.35 × Pugh score + 0.047 × age + 0.0045 × serum creatinine + (2.2 × form III) - 2.6, where ascites was scored as being absent, controlled with sodium restriction or diuretics or resistant to medical treatment (as scores 1, 2 or 3, respectively), and clinic-pathological form III (acute on top of chronic) was defined by the presence of at least one acute and one chronic feature and was coded as 1 for patients with form III and 0 for the other patients.

### Peer-review

This paper retrospectively evaluates whether various PIs are related to the one year survival of Egyptian patients with BCS in a single centre. The findings will be useful to clinicians treating these patients in Egypt.
